# Two-phase drift monitoring and automatic retraining (TPDM-AR) for electric load forecasting

**DOI:** 10.1016/j.mex.2026.103966

**Published:** 2026-05-20

**Authors:** Michel B. Oliveira, Leandro A. Silva

**Affiliations:** a Graduate Program in Electrical and Computer Engineering, Universidade Presbiteriana Mackenzie (Mackenzie Presbyterian University), Rua da Consolação 930, Consolação SP 01302-907, São Paulo, Brazil; b School of Computing and Graduate Program in Electrical and Computer Engineering, Universidade Presbiteriana Mackenzie (Mackenzie Presbyterian University), Rua da Consolação 930, Consolação SP 01302-907, São Paulo, Brazil

**Keywords:** Adaptive retraining, Concept drift, Data drift, Electric load forecasting, Kolmogorov–Smirnov windowing (KSWIN), Page–Hinkley (PH), Smart buildings

## Abstract

Monitoring incremental concept and data drift is important for electric load forecasting in smart buildings. This study proposes TPDM-AR (Two-Phase Drift Monitoring and Automatic Retraining), a customizable workflow that combines Adaptive Windowing (ADWIN), Kolmogorov–Smirnov Windowing (KSWIN), and Page–Hinkley (PH) drift detectors with a multilayer perceptron (MLP) regressor. Phase 1 monitors the raw active-power stream for distribution shifts, while Phase 2 confirms predictive degradation by tracking mean absolute error (MAE) using a smoothed MAE trajectory compared with a baseline reference threshold defined from a reference window. Retraining is initiated only when Phase 2 satisfies a consensus-and-persistence confirmation rule on the forecasting-error stream, while Phase 1 alerts on the raw series are reported as complementary signals. The method is validated on controlled synthetic linear and logarithmic drift scenarios and on the UCI Individual Household Electric Power Consumption dataset, reporting detector alert times and the corresponding evolution of forecasting error under the same monitoring setup.

• Two-phase drift monitoring that combines early change alerts with performance confirmation.
• Retraining is triggered only after Phase 2 consensus-and-persistence confirmation on MAE.
• Validated on synthetic drift and real-world electricity-demand data.

## Graphical abstract

**Figure:**
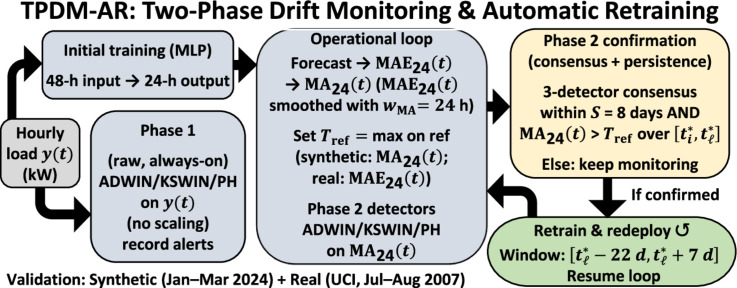

## Specifications table

**Table:** 

Subject area	Computer Science Computer Science
**More specific subject area**	Machine Learning, Concept Drift Detection, Time Series Forecasting.
**Name of your method**	Two-Phase Drift Monitoring and Automatic Retraining (TPDM-AR) for Electric Load Forecasting.
**Name and reference of original method**	Not applicable. TPDM-AR is a new method proposed in this article.
**Resource availability**	**Code & environment:** The files required to reproduce the reported outputs are provided in the Supplementary Material, including the main notebook/script (00_main_pipeline.ipynb), requirements.txt, requirements-lock.txt, and README.md. **Real‑world dataset:** UCI Individual Household Electric Power Consumption (https://doi.org/10.24432/C58K54). GitHub repository: The companion code is publicly available at https://github.com/MichelOliveira47/methodsx-drift-ml. The archived release corresponding to the accepted MethodsX version is available on Zenodo at https://doi.org/10.5281/zenodo.20338559. A fully reproducible implementation is also provided in the Supplementary Material.

## Background

Machine learning (ML) is widely used for power-demand (kW) forecasting to support energy management and sustainability in intelligent buildings and campuses [1,2]. In deployment, performance is challenged by drift: concept drift alters the statistical relationship between inputs and targets (P(Y|X)), whereas data drift shifts the input distribution (P(X)); both can degrade previously trained models [3,4]. Controlled synthetic evaluations also show that drift detectors can exhibit different alert patterns under changes in mean and standard deviation, motivating monitoring and confirmation rules that avoid unnecessary retraining when detector signals are not persistent [5].

In smart campuses, drift is common due to variable occupancy, seasonal demand, and heating, ventilation, and air conditioning (HVAC) operational variability, typically manifesting as incremental change and motivating adaptive monitoring. Over time, HVAC operation can undergo efficiency losses due to maintenance-related or mechanical factors (e.g., improper refrigerant charge, reduced airflow, and coil fouling), which tend to increase the electrical power and cooling energy required for the same thermal service. Such efficiency losses often manifest as gradual upward drifts in end-use power/energy, motivating adaptive monitoring [6,7].

Recent studies support this need: drift monitoring has been investigated for industrial load time series using long short-term memory (LSTM)-based neural networks, including continuous performance tracking [8]. Online drift-adaptive forecasting in energy domains has also been integrated into automated pipelines to mitigate performance degradation under changing conditions [9,10]. Drift detectors such as Adaptive Windowing (ADWIN), Kolmogorov–Smirnov Windowing (KSWIN), and Page–Hinkley (PH) can indicate changes either in the data stream or in error signals (e.g., mean absolute error, MAE) [11]. Alternative approaches based on modeling the forecasting error as a time series (e.g., autoregressive detectors) have also been proposed, and many real-world settings do not provide immediate ground-truth labels for online adaptation [12]. These methods are particularly relevant in batch-learning settings, where data arrive periodically and retraining may be constrained by infrastructure or regulations [9,10]. The River library provides implementations that support practical monitoring pipelines [13].

This work proposes the Two-Phase Drift Monitoring and Automatic Retraining (TPDM-AR) framework as a methodological contribution: a structured, customizable workflow for drift monitoring and retraining scheduling rather than a new algorithm. Its originality lies in a two-phase architecture that separates early warnings on the raw power stream (Phase 1) from performance-based confirmation on the forecasting-error stream (Phase 2), combined with a consensus-and-persistence rule for retraining decisions. A canonical multilayer perceptron regressor (MLPRegressor) illustrates that incremental drift can affect any regression model, while the framework remains model-agnostic and extensible to other scikit-learn-compatible regressors. Controlled synthetic drift profiles enable transparent evaluation of detector sensitivity and detection delay under known conditions, providing a reproducible proof of concept before real-world deployment. The objective is to show that the methodology functions as intended, not to benchmark TPDM-AR against alternatives or optimize its parameters.

## Method details

This study proposes TPDM-AR (Two-Phase Drift Monitoring and Automatic Retraining)**,** an experimental approach based on the generation of synthetic power-demand signals inspired by the typical air-conditioning usage profile at the Smart Campus of Mackenzie Presbyterian University. The objective is to evaluate, in a controlled manner, the ability to detect concept drift and data drift using multiple drift detectors coupled to a machine-learning forecasting model, and to trigger retraining according to the proposed two-phase decision rule.

### Definition of concept drift and data drift

In machine learning applied to time series, the data-generating process may change over time, which can affect the stability of model predictions. Data drift occurs when there is a change in the statistical distribution of the input variables (X), where t and t+1 denote consecutive time steps, as represented by [4]:

(1) $$P_{t}\left(X\right)\;\neq \;P_{t+1}\left(X\right)$$

This shift may occur alone (with a stable input–output relationship) or may co-occur with concept drift when the conditional relationship also changes. On the other hand, concept drift occurs when there is a change in the relationship between the input variables and the output variable, i.e., when the conditional distribution evolves over time [4]:

(2) $$P_{t}\left(Y\;|\;X\right)\neq \;P_{t+1}\left(Y\;|\;X\right)$$

Timely detection and mitigation of drift are required to maintain predictive accuracy and to prevent sustained performance degradation over time. Drifts can be abrupt (sudden change), incremental (slow and continuous change), gradual (transition with periods of uncertainty), or recurrent (previous concepts resurface).

This work focuses specifically on incremental drift, as it portrays slow distributional changes frequently observed in real electrical-load time series (hourly average active power demand), enabling detailed evaluation of detector responses [3,14].

### Virtual drift

In addition to data drift and concept drift, the literature also recognizes virtual drift (often treated as a special case of data drift), in which the input distribution changes while the underlying input–output relationship remains effectively unchanged: $P_{t}\left(X\right)\neq P_{t+1}\left(X\right)$ with $P_{t}\left(Y\;|\;X\right)\approx \;P_{t+1}\left(Y\;|\;X\right)$. In such cases, detectors may raise change alerts on the monitored stream, while forecast-error metrics (e.g., MAE) do not show sustained increases beyond baseline operating levels (i.e., no persistent degradation is observed) [15].

In this article, the term virtual drift is used in this operational (non-actionable) sense, whereas cases showing sustained performance degradation are treated as actionable drift without attempting to disentangle whether the cause is concept drift or impactful data drift (out-of-distribution operation).

### Synthetic signal generation

The dataset was synthetically generated using an empirically inspired function that simulates the hourly average active power y(t) (kW) as a function of the time of day. Let t denote the hourly time index of the synthetic series (one sample per hour). Let the hour of day at time index t be denoted by h(t)∈{0,1,…,23}. The hourly average active power at each time step is defined as:

### $y\left(t\right)=2.0+\;\xi_{t}$(3)

**Fig. 1:**
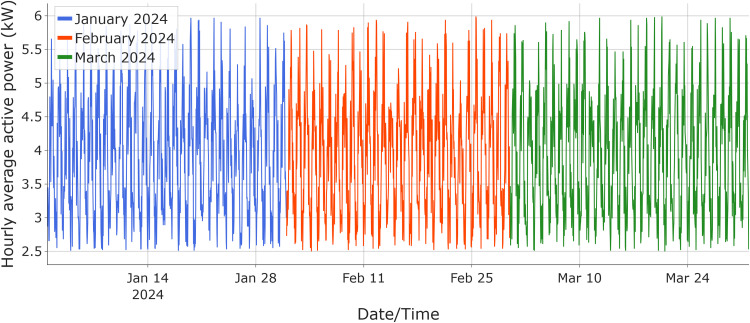
Visualization of synthetic hourly **average active-power (kW)** data generated for January, February, and March 2024.

The 2.0 kW term represents a constant baseline (base-load) component, while $\xi_{t}$​ is a random variable that follows a continuous uniform distribution whose parameters depend on the hour of the day h(t), defined as follows:

(4) $$\xi_{t}\;∼\;\left\{ \begin{array}{c} U\left(1.5,\;3.0\right)\;if\;6\leq h\left(t\right)<12\\ U\left(2.0,\;4.0\right)\;if\;12\leq h\left(t\right)<18\\ \begin{array}{c} U\left(1.0,\;2.5\right)\;if\;18\leq h\left(t\right)<22\\ U\left(0.5,\;1.5\right)\;otherwise \end{array} \end{array}\right.$$

where U(a,b) denotes a continuous uniform distribution over the interval [a,b]. All values of y(t) are rounded to two decimal places, reflecting the resolution of metered active power readings; the baseline series is shown in Fig. 1 and is deterministically reproducible using a fixed random seed (NumPyseed=42); the corresponding Supplementary Synthetic Dataset is provided in the Supplementary Material.

The intervals for each period of the day seek to reproduce the real variability observed in HVAC systems, ensuring that synthetic data are challenging and suitable for evaluating prediction methods and drift detection [16]. Three full months of synthetic data were generated (January–March 2024).

### Controlled drift insertion

As air-conditioning/heat pump (AC/HP) performance deteriorates over time (e.g., due to improper refrigerant charge, deficient airflow, and coil fouling), maintaining the same thermal service typically requires higher electrical input, increasing cooling energy use [6]. Accordingly, the synthetic series was perturbed with a controlled, gradually increasing drift during March to emulate progressive increases in active-power demand**.**

After generating the synthetic hourly load series, two forms of incremental drift were injected during March to emulate gradual changes in the active power demand pattern. Let t denote the hourly index adopted in the previous section. For the month of March, each hourly sample is equivalently represented by the pair (d,h), where d∈{1,…,31} denotes the day of the month and h∈{0,…,23} denotes the hour of the day, with the mapping t=24(d−1)+h. Let $y_{d,h}$ be the original hourly average active power (load), and $y_{d,h}^{\prime }$the drifted value after distortion. The linear drift is defined as:

(5) $$y_{d,h}^{{}^{\prime }}=\;y_{d,h}\;\cdot \;\left[1+\;\frac{p}{100}\;\cdot \;\frac{d-1}{30}\;\right]$$

The logarithmic drift is defined as:

(6) $$\;y_{d,h}^{{}^{\prime }}=\;y_{d,h}\;\cdot \;\left[1+\;\frac{p}{100}\;\cdot \;\frac{\ln d}{\ln31}\;\right]$$

where p∈{10,20,30,40,50} is the final percentage increase applied by the end of the month, and ln(·) denotes the natural logarithm. By the change-of-base identity, $\ln\left(d\right)/\;\ln\left(31\right)=\text{lo}g_{31}\left(d\right)$ which increases from 0 to 1 for d∈[1,31] and therefore imposes a logarithmically increasing multiplicative distortion on $y_{d,h}$, yielding $y_{d,h}^{\prime }$. In both cases, the distortion is null at the beginning of the month (d=1) and reaches its maximum value p% on the last day (d=31). Thus, the first non-zero distortion occurs from day d=2 onward and increases incrementally (i.e., non-decreasing) until d=31.

These controlled distribution shifts enable systematic evaluation of detector sensitivity and detection delay under different drift severities, and allow assessment of whether the injected input changes produce persistent degradation in forecasting performance.

### Machine learning model training and evaluation

For the synthetic dataset, January 2024 is reserved exclusively for training the forecasting model. For the real-world dataset, the model is trained on July 2007 and deployed for monitoring in August 2007. In this method, an MLPRegressor (Multilayer Perceptron for Regression) [17] was configured as shown in Table 1.

**Table 1:** Hyperparameters of the MLPRegressor and their justifications (scikit-learn implementation).

Hyperparameter	Value	Justification
hidden_layer_sizes	(64, 64)	Sufficient for capturing short-term nonlinear patterns.
activation	ReLU	Empirical default in many regression baselines; widely used in energy-forecasting MLPs.
solver	Adam	First-order optimizer widely used for MLP training; suitable for non-convex objectives.
max_iter	1000	Ensures convergence within practical CPU runtimes in these experiments.
random_state	42	Reproducibility.

**Fig. 2:**
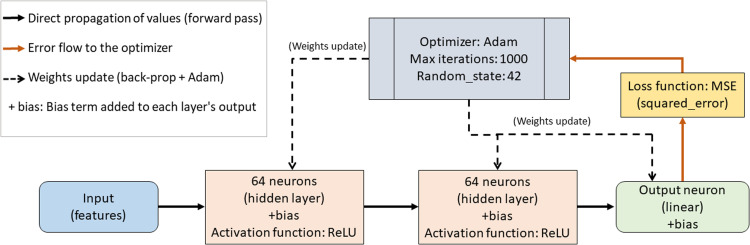
Simplified architecture of the MLPRegressor used in the experiment (scikit-learn).

All remaining MLPRegressor hyperparameters not listed in Table 1 follow scikit-learn defaults; the complete configuration used to reproduce the experiments is provided in the Supplementary Material. Fig. 2 presents the simplified architecture of the MLPRegressor adopted. The model takes 48-h sliding windows as input and processes them into two hidden layers with 64 neurons each, activated by ReLU and with bias terms.

The MLP uses a linear output layer (identity activation) to produce the next 24-step-ahead (24-h) forecast horizon. The training uses the Adam optimizer, with up to 1000 iterations and random_state = 42 for reproducibility. The mean squared error (MSE) loss function guides backpropagation and weight updates. The diagram highlights the direct flow, error propagation, and weight updating during learning.

The MLPRegressor was used as a baseline forecasting model to capture potential nonlinear relationships in the load series and to generate a consistent error stream for drift monitoring. Training was performed with the Adam optimizer under a fixed set of hyperparameters, and the monitoring signal was derived from the multi-step forecast errors using a 24-h moving average ($MA_{24}\left(t\right)$), as defined in this workflow [18].

Input data consists of 48-h sliding windows $\left(x_{i}=\;\left(y_{i},\ldots ,y_{i+47}\right)\right)$ with the target being the forecast of the subsequent 24-h horizon $\left(z_{i}=\;\left(y_{i+48},\ldots ,y_{i+71}\right)\right)$. MinMaxScaler is fitted on the baseline training month (synthetic: January 2024; real-world: July 2007) (feature_range=[0,1]). Values outside the baseline range may map outside [0,1] during monitoring; this is expected and does not cause leakage. The equation used for normalization is given by [17]:

(7) $$x_{\text{norm}}=\;\frac{x-\;x_{\text{min}}}{x_{\text{max}}-\;x_{\text{min}}}$$

where x represents the original active power value; $x_{min}$​ and $x_{max}$​ are, respectively, the lowest and highest active power values observed in the baseline training month (synthetic: January 2024; real-world: July 2007), and $x_{norm}$​ is the corresponding normalized value. The month of February is used as a reference for the test, characterizing a period of baseline (no-drift) operation. In turn, the month of March, with artificially injected (synthetic/forced) drift, is used to evaluate the model’s performance under drift-injected conditions [3].

### Application of drift detectors

Three online drift detectors (ADWIN, KSWIN, and PH) from the River library were used. The main configuration parameters for each detector are summarized in Table 2.

**Table 2:** Configuration of drift detectors used in the experiments (River library).

Detector	Statistics	Key parameters in the experiment
ADWIN	Maintains 2 adaptive windows; tests whether ∣μW − μW′∣> ε (Hoeffding Bound)	significance delta $(\delta_{\text{ADWIN}})=0.8$
KSWIN	KS-test incremental; compares an r-size sub-sample with sliding window $w_{KS}$	alpha(α)=0.2, stat_size (r)=24, window_size $(w_{KS})=48$
PH	$m_{t}=\sum_{i=1}^{t}\left(x_{i}-{\hat{\mu }}_{i}-\delta \right)$; drift if $m_{t}$− $\text{mi}n_{k\leq t}$($m_{k}$) > λ	min_instances=24, tolerance delta $\left(\delta_{\text{PH}}\right)=\;10^{-4}$, threshold (λ)=30

Detector parameters were selected empirically to match the study’s temporal granularity (48-h input windows and a 24-h forecast horizon), using the drift-free reference month (February) to control the Phase 1 false-alert rate. For ADWIN, the significance parameter $\delta_{\text{ADWIN}}$ was swept from $10^{-4}$ to 0.8; $\delta_{\text{ADWIN}}=0.8$ (the largest value yielding zero alerts in February) was selected to preserve sensitivity to mild drift; this value is dataset- and calibration-specific and is not intended as a default ADWIN setting. For KSWIN, a grid search over α ∈ [10^–3^, 0.9], *r* ∈ {12, 24, 48}, and *w_KS_* ∊ {24, 48, 72} led to *α =* 0.2, *r* = 24, and *w_KS_* = 48, also yielding zero alerts in February while producing sparse triggers under sustained shifts in the synthetic scenarios. For pH, λ ∈ {10, 20, 30, 50} was evaluated and λ = 30 was selected; $\delta_{\text{PH}}$ was set to $10^{-4}$ based on the drift-free reference month (February) to control the Phase 1 false-alert rate under the stream characteristics of this study. Drift detectors are applied in two phases: Phase 1 monitors the raw stream, whereas Phase 2 monitors a smoothed model-error stream. Phase 1 alerts are treated as preliminary signals; retraining is initiated only when the Phase 2 persistence criterion (defined later in this section) is satisfied.

For PH, min_instances=24 ensures that at least one full day of observations is available before an alert can be raised. These settings are context-dependent rather than globally optimal, but provide a practical starting point for hourly average active-power (load) data with similar noise characteristics and can be refined to match the desired alert frequency. When transferring TPDM-AR to other datasets or deployments, detector hyperparameters should be recalibrated for the sampling rate, forecast horizon, error-signal construction, and target alerting behavior rather than reused unchanged.

Note: Parameter names follow the River API; identical names (e.g., delta) are detector-specific and have different meanings across methods ($\delta_{\text{ADWIN}}$: significance; $\delta_{\text{PH}}$: tolerance).

**Adaptive Windowing (ADWIN):** ADWIN maintains an adaptive window and evaluates candidate splits into two sub-windows W and $W^{\prime }$. Drift is signaled when the difference between the sample means exceeds a Hoeffding-bound threshold [16]:

(8) $$\left|\mu_{W}\;-\mu_{W^{\prime }}\right|\;>\epsilon ,\;\epsilon =\;\epsilon \left(\delta ,\left|W\right|,\left|W^{\prime }\right|\right)$$

where $\mu_{W}$ and $\mu_{W^{\prime }}$ are the sample means of Wand $W^{\prime }$, respectively; |W| and $|W^{\prime }|$ are their sizes; ϵ is the Hoeffding-bound detection threshold determined by δ and the sub-window sizes [19].

**Kolmogorov–Smirnov Windowing (KSWIN)**: KSWIN incrementally performs a Kolmogorov–Smirnov test between a recent sub-sample of size r and a sliding window of size $w_{\text{KS}}$. With each new observation, it updates the empirical cumulative distribution functions (ECDFs) of both samples and computes the test statistic as follows [16]:

(9) $$D=\;\text{su}p_{x}\left|F_{s\text{ub}}\left(x\right)-F_{\text{window}}\left(x\right)\right|$$

where $F_{s\text{ub}}$ is the ECDF of the most recent r points and $F_{\text{window}}$​ is the ECDF of the last $w_{\text{KS}}$ points. If D exceeds the critical value defined by the significance level α, the detector signals that drift has occurred. In River, this comparison is made incrementally with each new piece of data, with the parameters α**,** r, and $w_{\text{KS}}$ controlling, respectively, the significance level, the sub-sample size, and the size of the main window [20].

**Page–Hinkley (PH):** The PH method monitors the accumulated deviation of the stream from an estimated mean ${\hat{\mu }}_{i}$ [21]:

(10) $$m_{t}=\;\sum_{i=1}^{t}\left(x_{i}-{\hat{\mu }}_{i}-\delta \right),\;\text{drift}\;\text{if}m_{t}-\text{mi}n_{k\leq t}\left(m_{k}\right)>\lambda$$

Here, t denotes the current time step, i indexes the observations accumulated up to t, and k indexes past time steps used to evaluate the historical minimum. At each time step, the cumulative statistic $m_{t}$ and its historical minimum $\text{mi}n_{k\leq t}\left(m_{k}\right)$ are tracked. When $m_{t}-\text{mi}n_{k\leq t}\left(m_{k}\right)$ exceeds λ, a change in the mean level of the stream is signaled. In the experiment, min_instances delays detection to reduce early false alarms, and δ prevents small fluctuations from being interpreted as drift (Table 2) [22].

In this method, the detectors (ADWIN, KSWIN, and PH) are applied to two streams: (i) directly on the active-power (load) series (to identify data drift) and (ii) on an MAE-based error stream derived from the MLP forecasts (to identify performance degradation as evidence of actionable drift). The exact construction of this error stream is detailed in the two-phase drift monitoring logic subsection**.** The MAE is calculated as follows [7]:

(11) $$\text{MAE}=\frac{1}{n}\sum_{t=1}^{n}\left|y_{t}-\;{\hat{y}}_{t}\right|$$

where $y_{t}$ is the actual observed value at time t, ${\hat{y}}_{t}$ is the value predicted by the model, and n is the number of observations. Although Mean Squared Error (MSE) and Root Mean Squared Error (RMSE) are also computed and reported as supplementary diagnostics (e.g., in Supplementary Tables/Figs and the exported workbooks), the TPDM-AR monitoring and decision logic rely exclusively on MAE. This combined approach allows the detection of not only changes in data distribution but also degradations in model performance, signaling the need to trigger retraining [8].

### Two-phase drift monitoring logic (Phase 1 and Phase 2)

In this study, drift monitoring follows a two-phase logic. **Phase 1 (raw-series screening):** ADWIN, KSWIN, and PH are applied directly to the raw (non-normalized) hourly active-power series to log distribution shifts in the monitored stream (i.e., data-drift signals). Because distribution shifts do not necessarily translate into actionable predictive degradation, Phase 1 alerts are interpreted as evidence of raw-series shifts. When Phase 2 does not confirm sustained performance loss, these Phase 1 alerts are interpreted as consistent with a virtual-drift condition (i.e., a shift without persistent predictive degradation). Phase 1 alerts are complementary and may not always precede Phase 2, since Phase 2 monitors the induced forecasting-error stream.

**Phase 2 (error-based confirmation):** The same detectors are applied to an MAE-based error stream derived from the forecasting model to confirm sustained predictive degradation. The Phase 2 monitoring signal and its threshold calibration are defined differently in the synthetic experiments and in the real-data validation to reflect the distinct evaluation protocols, while preserving the same confirmation logic.

**Synthetic experiments (hourly pointwise error stream)**: In the synthetic regime, the pointwise absolute error at hour t is computed on denormalized values as $\varepsilon \left(t\right)=|y\left(t\right)-\hat{y}\left(t\right)|$, where y(t) denotes the observed value and $\hat{y}\left(t\right)$ the model forecast at hour t. Phase 2 monitors a smoothed MAE-based error series obtained as a moving average of ε(t) with window length $w_{\text{MA}}=24$​ (in hours), defined as:

(12) $${\text{MAE}}_{smooth}^{\left(w_{\text{MA}}\right)}\left(t\right)=\;\frac{1}{w_{\text{MA}}}\sum_{k=0}^{w_{\text{MA}}-1}\varepsilon \left(t-k\right)$$

where $w_{\text{MA}}$​ is the moving-average window length (hours) and t indexes the hourly timeline. In the synthetic regime, ε(t) is the pointwise absolute error and $MA_{24}\left(t\right)$ is its 24-h moving average ($w_{\text{MA}}=24$). When $w_{\text{MA}}=24$, the smoothed series is denoted $MA_{24}\left(t\right)$ and is the Phase 2 signal used in the validation experiments.

A Phase 2 reference threshold, denoted $T_{\text{ref}}$​, is defined from a drift-free reference period ref using a conservative max rule:

(13) $${\text{MAE}}_{\text{max}}^{\text{ref}}=\max_{t\;\in \;\text{ref}}{\text{MAE}}_{\text{smooth}}^{\left(w_{\text{MA}}\right)}\left(t\right)\;$$

In the synthetic experiments, $T_{\text{ref}}$​ is set to ${\text{MAE}}_{\text{max}}^{\text{ref}}$​, computed as the maximum of the smoothed monitoring signal $MA_{24}\left(t\right)$ over the drift-free baseline month (February). Because February is controlled and guaranteed drift-free, ${\text{MAE}}_{\text{max}}^{\text{ref}}$​ is computed over the full month to capture the complete weekly variability of $MA_{24}\left(t\right)$ under stability.

**Real-data validation (horizon-aggregated** $\mathrm{MA}E_{24}\left(t\right)$ **indexed at the forecast origin):** In real-data validation, a 24-step forecast is issued at each forecast-origin time t using a 48-h input window, producing ${\hat{y}}_{t+h|t}$​ for h=1,…,24. The corresponding absolute errors are $AE_{t+h|t}=\left|y_{t+h}-{\hat{y}}_{t+h|t}\right|$, h=1,…,24 and they are aggregated into a scalar error per forecast origin:

(14) $$\text{MA}E_{24}\left(t\right)=\frac{1}{24}\sum_{h=1}^{24}AE_{t+h|t}\;$$

The Phase 2 monitoring signal is then obtained by smoothing the $\text{MA}E_{24}\left(t\right)$ stream using the same moving-average definition in (12) with $w_{\text{MA}}=24$, yielding $MA_{24}\left(t\right)$. Hereafter, $\text{MA}E_{24}\left(t\right)$ denotes the horizon-aggregated MAE at forecast origin t, and $MA_{24}\left(t\right)$ denotes the 24-sample moving-average smoothing of $\text{MA}E_{24}\left(t\right)$ ($w_{\text{MA}}=24$). For real measurements, the default reference threshold is calibrated on a short reference window ref (the set of forecast-origin timestamps t in an initial 7-day reference week) using the non-smoothed $\text{MA}E_{24}\left(t\right)$ stream. In real data, this initial window is a pragmatic calibration period; drift-free stationarity is not assumed beyond it:

(15) $${\text{MAE}}_{\text{max}}^{\text{ref}}=\max_{t\;\in \;\text{ref}}\text{MA}E_{24}\left(t\right),\;T_{\text{ref}}={\text{MAE}}_{\text{max}}^{\text{ref}}\;$$

After retraining and redeployment, $T_{\text{ref}}$​ can be recomputed for the next cycle (dynamic per cycle), or kept fixed over the model lifetime (fixed global). In this study, the fixed-global policy is adopted. Detectors are applied to $MA_{24}\left(t\right)$ for Phase 2 monitoring, while calibrating $T_{\text{ref}}$​ on non-smoothed $\text{MA}E_{24}\left(t\right)$ preserves the nominal peak envelope that could be attenuated by smoothing. This asymmetric design is intentionally conservative: it avoids underestimating $T_{\text{ref}}$​ due to smoothing, thereby reducing the likelihood of premature Phase 2 confirmation.

**Rationale for raw reference and smoothed monitoring:** The reference window is a controlled calibration interval and is treated as a pragmatic baseline (assumed nominal) for defining $T_{\text{ref}}$​. Threshold calibration is performed on the non-smoothed $\text{MA}E_{24}\left(t\right)$ so that the threshold reflects the upper envelope of error realizations observed under nominal conditions; applying a moving average at this stage may attenuate peaks and yield a lower threshold.

After deployment, $\text{MA}E_{24}\left(t\right)$ can present high-frequency variability due to measurement noise, operating-point changes, and short-lived events. For this reason, monitoring and detector operation are performed on $MA_{24}\left(t\right)$, obtained by smoothing $\text{MA}E_{24}\left(t\right)$ with $w_{\text{MA}}=24$, so that Phase 2 confirmation is driven by sustained deviations rather than isolated spikes.

This yields the following practical distinction while preserving the same Phase 2 logic:

• Synthetic regime: the drift-free reference month is controlled and reliably stable, enabling a conservative threshold based on the maximum of the smoothed monitoring signal over the entire month.
• Real-data regime: long-span stationarity cannot be assumed; therefore, the threshold is calibrated from a short, initial reference week, using a non-smoothed error scalar to avoid masking worst-case error realizations.

**Reference threshold calibration and optional modes:** For real measurements, drift-free stationarity cannot be assumed over long spans; therefore, the reference threshold is calibrated using only an initial 7-day reference week immediately after deployment (Aug 1–7), reducing the risk of contaminating the threshold with unobserved regime changes later in the month.

**Default (conservative max rule, used in all reported results):** $T_{\text{ref}}={\text{MAE}}_{\text{max}}^{\text{ref}}$ where ${\text{MAE}}_{\text{max}}^{\text{ref}}$​ is computed over the reference window (synthetic: full February on $MA_{24}\left(t\right)$; real: reference week on $\text{MA}E_{24}\left(t\right)$, with monitoring performed on $MA_{24}\left(t\right)$).

**Optional threshold modes (provided as configuration hooks; not validated in this study):**

• Quantile-based threshold: $T_{\text{ref}}=Q_{0.95}\left({\left\{MA_{24}\left(t\right)\right\}}_{t\;\in \;\text{ref}}\right)$, where $Q_{0.95}\left(\cdot \right)$ denotes the 95th percentile computed over the reference window ref.
• Parametric threshold: $T_{\text{ref}}=\mu_{\text{ref}}+\kappa \sigma_{\text{ref}}$​, where $\mu_{\text{ref}}$​ and $\sigma_{\text{ref}}$​ are the mean and standard deviation of $MA_{24}\left(t\right)$ within the reference window, and κ is a user-defined sensitivity factor (κ=3 by default).

These optional modes are not validated in the present study and are included only as extensibility options; all reported results adopt the conservative default $T_{\text{ref}}={\text{MAE}}_{\text{max}}^{\text{ref}}$​.

### Phase 2 confirmation and retraining trigger (consensus and persistence criteria)

The consensus-and-persistence rule operationalizes a deliberate design principle: retraining should be triggered only when multiple independent detectors agree, referred to here as consensus, and when the degradation signal is sustained over time rather than transient, referred to here as persistence. This principle is grounded in established findings showing that individual drift detectors can produce false alerts in response to non-actionable fluctuations, and that requiring signal persistence substantially reduces unnecessary retraining [5,11]. The consensus condition reduces susceptibility to single-detector false positives by requiring agreement across ADWIN, KSWIN, and PH, which are three detectors with distinct statistical sensitivities, within a bounded time window S. The persistence condition additionally guards against retraining triggered by short-lived error excursions that recover without intervention. Together, these conditions implement a conservative confirmation logic designed for batch-learning settings where retraining has non-negligible operational cost [9,10]. The specific parameter values, namely S=8 days and $T_{\text{ref}}$ calibrated as the conservative maximum over the reference window, were set empirically, guided by the dataset characteristics and the referenced literature. A formal sensitivity analysis is identified as future work.

• Consensus condition: Each detector (ADWIN, KSWIN, PH) must trigger at least once on $MA_{24}\left(t\right)$, within a bounded interval $\left[t_{i}^{*},\;t_{l}^{*}\right]$, where $t_{i}^{*}$ is the first Phase 2 alert and $t_{l}^{*}$ is the alert that completes the three-detector consensus. The interval must satisfy $t_{l}^{*}-t_{i}^{*}\leq S$ days.
• Persistence condition: The monitored signal must remain above threshold throughout the consensus interval; $MA_{24}\left(t\right)>T_{\text{ref}}$, $\forall \;t\;\in \;[t_{i}^{*},\;t_{l}^{*}]$.
• Trigger: A retraining action is initiated only when consensus and persistence are simultaneously satisfied.

**Retraining window and execution:** When confirmation is achieved at $t_{l}^{*}$​, the retraining dataset is selected using a fixed-length window anchored at consensus completion, [$t_{l}^{*}-22$ days, $t_{l}^{*}+7$ days], yielding approximately one calendar month of data. Because the window includes a 7-day post-consensus buffer, retraining is executed only after the required post-consensus observations become available. In TPDM-AR, “automatic retraining” denotes automatic rule-based scheduling after Phase 2 confirmation rather than immediate model refitting at the first detector alert.

This choice preserves a training horizon comparable to the original one-month baseline, emphasizes recency under non-stationarity, and constrains the verification latency required to obtain post-consensus ground-truth observations for error-based confirmation and retraining [11,23,24]**.**

### Operational summary

TPDM-AR is a two-phase monitoring pipeline for hourly active-power (load) streams that combines an MLP-based multi-step forecasting model with three statistical drift detectors (ADWIN, KSWIN, and PH). Phase 1 applies the detectors to the raw series to log distribution-shift alerts. Phase 2 assesses performance degradation on an MAE-derived monitoring signal and triggers retraining only when a consensus-and-persistence confirmation rule is satisfied. The full executable procedure, including the synthetic versus real-data handling and the exact threshold calibration and retraining windowing, is provided below. In particular, Phase 2 uses two regime-specific error constructions: an hourly pointwise absolute-error stream ε(t) computed on denormalized values from an unfolded multi-step forecast in the synthetic experiments, and a horizon-aggregated $\text{MA}E_{24}\left(t\right)$ indexed at the forecast origin in real-data validation.

### Step-by-step procedure (TPDM-AR replication guide)

**Step 1**. Define the monitoring setup (streams and constants): hourly load series y(t); forecasting design (48-h input → 24-h horizon); detectors {ADWIN, KSWIN, PH}; Phase 2 smoothing window $w_{\text{MA}}=24$; and consensus horizon S=8 days.

**Step 2.** Prepare the load data y(t) as an hourly series.

• Synthetic validation: generate Jan–Mar 2024 and keep Jan for training, Feb as drift-free reference, and Mar for drift-injected evaluation.
• Real-data validation: build the hourly series y(t) from the UCI Global_active_power (kW) by parsing Date and Time into timestamps, handling missing values via time interpolation, and resampling to hourly mean active power. Define the initial reference window ref as 7 consecutive days (Aug 1–7), used later to calibrate the reference threshold in Step 10.

**Step 3.** (Synthetic only) Inject controlled incremental drift in March using linear and logarithmic distortion profiles with final intensity p∈{10,20,30,40,50} % by day 31.

**Step 4.** Build supervised learning samples with sliding windows: inputs $x_{i}=\left(y_{i},\ldots ,y_{i+47}\right)$ and targets $z_{i}=\left(y_{i+48},\ldots ,y_{i+71}\right)$.

**Step 5.** Fit the scaler on the training baseline month only (synthetic: January 2024; real-world: July 2007) and normalize both inputs and outputs using MinMaxScaler [0,1] to avoid leakage.

**Step 6.** Train the forecasting model (MLPRegressor) on the baseline month only (synthetic: January 2024; real-world: July 2007), freeze the model, and keep the detector hyperparameters fixed (Table-based configuration).

**Step 7.** Run multi-step forecasting during monitoring: at each forecast origin t, issue a 24-step ahead forecast ${\hat{y}}_{t+h|t}$, h=1,…,24, and create a consistent error stream for monitoring.

• Synthetic regime (to form a single-valued hourly forecast stream $\hat{y}\left(t\right)$): issue one 24-step forecast per daily anchor (e.g., 00:00). This avoids overlapping horizons across multiple forecast origins and yields exactly one forecast per hour for ε(t). Then unfold the horizon to assign ${\hat{y}}_{t+h}\leftarrow {\hat{y}}_{t+h|t}$ for h={1,…,24}. Concatenate daily horizons to obtain the hourly $\hat{y}\left(t\right)$ stream used to compute ε(t).
• Real-data regime: keep the per-origin multi-step forecasts ${\hat{y}}_{t+h|t}$​ (one vector per forecast origin t; in this study, forecast origins are hourly (∆*t* = 1 h), i.e., one forecast per hour) to build the horizon-aggregated error $\text{MA}E_{24}\left(t\right)$.

**Step 8.** Compute the Phase 2 “raw” error stream (regime-specific):

• Synthetic regime: compute the hourly pointwise absolute error on denormalized values (after inverse-transform) as $\varepsilon \left(t\right)=|y\left(t\right)-\hat{y}\left(t\right)|$.
• Real-data regime: compute $AE_{t+h|t}=\left|y_{t+h}-{\hat{y}}_{t+h|t}\right|$ and aggregate it into $\text{MA}E_{24}\left(t\right)=\frac{1}{24}\sum_{h=1}^{24}AE_{t+h|t}$​ (one scalar per forecast origin).

**Step 9.** Build the Phase 2 monitoring signal by smoothing the regime-specific raw error stream (synthetic: ε(t); real-data: $\text{MA}E_{24}\left(t\right)$) with a moving average $w_{\text{MA}}=24$, yielding $MA_{24}\left(t\right)$. Apply detectors in Phase 2 on the smoothed signal to emphasize sustained deviations.

• Synthetic regime: smooth ε(t) using $w_{\text{MA}}=24$ to obtain $MA_{24}\left(t\right)$ as in Eq. (12).
• Real-data regime: smooth $\text{MA}E_{24}\left(t\right)$ using the same definition to obtain $MA_{24}\left(t\right)$.

**Step 10.** Calibrate the Phase 2 reference threshold $T_{\text{ref}}$​ on an initial reference interval, using the regime-appropriate monitoring scalar.

• Synthetic regime: Since February is drift-free by construction, compute $T_{\text{ref}}$​ over the full month as $T_{\text{ref}}=\text{ma}x_{t\;\in \;\text{Feb}\;}MA_{24}\left(t\right)$.
• Real-data regime: Calibrate on a short initial reference week ref (7 consecutive days from the start of monitoring), using the horizon-aggregated error indexed at the forecast origin, as $T_{\text{ref}}=\text{ma}x_{t\;\in \;\text{ref}}\;\text{MA}E_{24}\left(t\right)$. Use either a fixed-global $T_{\text{ref}}$​ or per-cycle updating (this study uses fixed-global). For conservatism, $T_{\text{ref}}$​ is computed on non-smoothed $\text{MA}E_{24}\left(t\right)$ although detectors operate on smoothed $MA_{24}\left(t\right)$.

Note: The subscript “24″ appears in both $\text{MA}E_{24}\left(t\right)$ (24-step horizon aggregation) and $MA_{24}\left(t\right)$ (24-sample moving average), but they refer to different operations: horizon aggregation versus temporal smoothing.

**Step 11.** Phase 1 monitoring (raw stream): run ADWIN/KSWIN/PH on the raw y(t) stream and log Phase 1 alerts (reporting may include a Daily-MAE curve as context).

**Step 12.** Phase 2 monitoring (impact stream): run the same detectors on $MA_{24}\left(t\right)$, log alert timestamps, and form the candidate consensus interval $\left[t_{i}^{*},\;t_{l}^{*}\right]$ from the first Phase 2 alert to the alert that completes three-detector consensus.

**Step 13.** Apply the Phase 2 confirmation rule: confirm degradation only if (i) three-detector consensus occurs within horizon S, and (ii) the persistence condition holds, i.e., $MA_{24}\left(t\right)>T_{\text{ref}}$ for all $t\in [t_{i}^{*},\;t_{l}^{*}]$. Here, the consensus completion time $t_{l}^{*}$​ is defined as the timestamp of the third detector’s first alert (within the window S).

**Step 14.** If confirmed, schedule retraining using the fixed-length window anchored at the consensus completion time [$t_{l}^{*}-22$ days, $t_{l}^{*}+7$ days]; execute retraining after the +7-day post-consensus buffer becomes available. After retraining and deployment, start a new monitoring cycle by reinitializing ADWIN, KSWIN, and PH for Phase 1 and Phase 2. The new cycle uses a 7-day reference week to define the Phase-2 baseline, and monitoring resumes at the subsequent stream start; if configured, $T_{\text{ref}}$ is recalibrated for the new cycle (or retained under a fixed-global policy).

**Step 15.** Save reproducibility artifacts: configuration (all hyperparameters), timestamps of Phase 1/Phase 2 alerts, error streams ($\text{MA}E_{24}\left(t\right)$, $MA_{24}\left(t\right)$), and validation metrics (MAE/MSE/RMSE), plus figures and logs required to reproduce the exact runs.

## Method validation

### Synthetic signal

Drift intensities of 10 %–50 % were evaluated for both linear and logarithmic scenarios to validate TPDM-AR under controlled incremental drift. To avoid repetition and improve conciseness, the main figures illustrate only the 50 % drift level, which corresponds to the highest intensity. Complete quantitative outputs for all drift levels, including MAE as the primary metric along with MSE and root mean squared error (RMSE) as complementary metrics, are provided in Supplementary Table S1 (worksheets Lin10–Lin50 and Log10–Log50).

### Linear drift

**Fig. 3:**
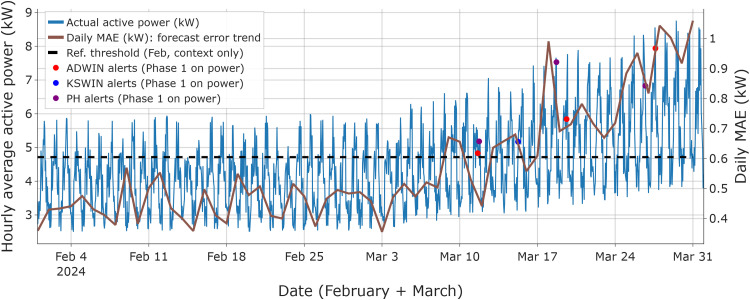
Phase 1 monitoring under **incremental linear drift (final level: 50 %)**: ADWIN, KSWIN, and PH alerts overlaid on the raw hourly active-power series, with the daily MAE trend. The dashed line indicates the February-based reference threshold, displayed only as a coarse reference against the daily error trend and not used for decision-making in this figure.

Fig. 3 summarizes the 50 % linear-drift scenario by overlaying the raw hourly active-power series with a daily MAE curve, computed as the day-wise mean of the absolute forecast error $\varepsilon \left(t\right)=|y\left(t\right)-\hat{y}\left(t\right)|$, to highlight the overall forecast error trend. The dashed line shows the February baseline MAE threshold for context only. In February (drift-free segment), no detector alerts were recorded.

After the non-zero drift starts on March 2, Phase 1 raises the first alerts on March 11 (ADWIN and PH), followed by KSWIN on March 15. Supplementary Fig. S1 reports the corresponding prediction-vs-measurement traces used to validate the Phase 2 error-stream behavior.

**Fig. 4:**
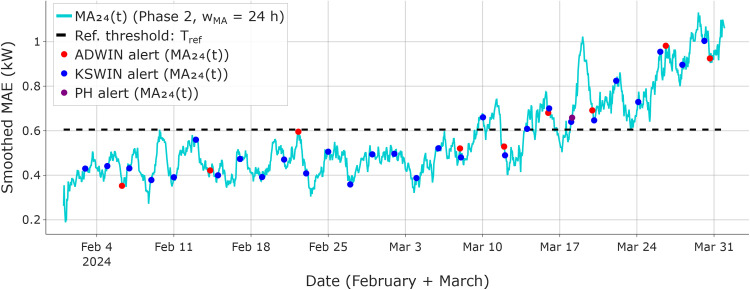
Phase 2 drift confirmation (50 % linear drift): the 24-h smoothed MAE signal is monitored by ADWIN, KSWIN, and PH, with alerts overlaid on the smoothed curve. The dashed line indicates the reference threshold, calibrated on the drift-free reference window and used as the Phase 2 decision boundary when evaluating persistence during the consensus interval.

Phase 2 is summarized in Fig. 4, where ADWIN, KSWIN, and PH are applied to the smoothed MAE series $MA_{24}\left(t\right)$, using a reference threshold $T_{\text{ref}}$.

In this stream, Phase 2 alerts are logged between March 10–18 (KSWIN on March 10; ADWIN on March 15; PH on March 18), completing the three-detector consensus. Accordingly, the consensus interval $\left[t_{i}^{*},\;t_{l}^{*}\right]$ is defined as March 10–18, with $t_{i}^{*}=\text{March}\;10$​ and $t_{l}^{*}=\text{March}\;18$. The persistence criterion is satisfied over this interval, with $MA_{24}\left(t\right)>T_{\text{ref}}$​ for all $t\;\in \;[t_{i}^{*},\;t_{l}^{*}]$ ($T_{\text{ref}}=0.605\;\text{kW}$).

As a validation check of the Phase 2 monitoring signal computation, Fig. 4 shows the computed $MA_{24}\left(t\right)$ reaching a maximum of ≈1.13kW (March 29) and ending at ≈1.06kW (March 31) for the 50 % linear-drift example. To preserve the original training design (one month of data), the retraining window is defined as [$\;t_{l}^{*}-22$ days, $t_{l}^{*}+7$ days], yielding February 25–March 25 for $t_{l}^{*}=\text{March}\;18$. This window incorporates the shifted load regime while retaining representative pre-drift samples.

### Logarithmic drift

**Fig. 5:**
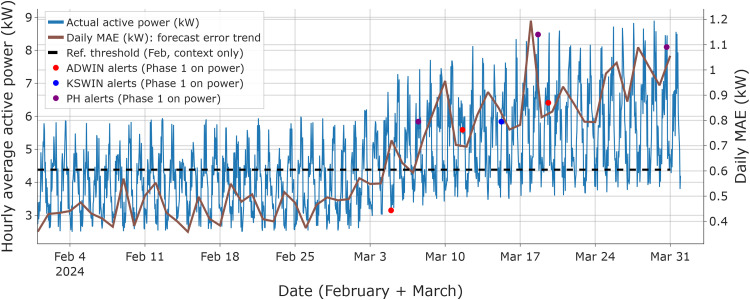
Phase 1 monitoring under incremental logarithmic drift (final level: 50 %): ADWIN, KSWIN, and PH alerts are overlaid on the raw hourly active-power series (Feb: drift-free; Mar: drift). The daily MAE curve is shown as a compact error-trend context, and the dashed line indicates the February-based reference threshold​, displayed only as a coarse reference against the daily error trend and not used for decision-making in this figure.

Fig. 5 summarizes the 50 % logarithmic-drift scenario by combining Phase 1 alerts on the raw (non-normalized) active-power series with the daily MAE trajectory.

The daily MAE is computed as the day-wise mean of the hourly absolute forecast error, $\varepsilon \left(t\right)=|y\left(t\right)-\hat{y}\left(t\right)|$, providing a compact validation view of (i) Phase 1 distribution-change alerts on the raw input stream and (ii) the corresponding forecasting-error trend. The dashed line indicates the February-based reference threshold, $T_{\text{ref}}=0.605\;\text{kW}$.

With controlled logarithmic drift injected in March, Phase 1 alerts are logged on March 4 (ADWIN), March 7 (PH), and March 15 (KSWIN), with additional alerts on March 11 (ADWIN) and March 18 (PH). In parallel, the daily MAE increases after early March and frequently exceeds the February baseline ($T_{\text{ref}}$) later in the month. Supplementary Fig. S2 provides the corresponding prediction-versus-measurement traces (with and without injected drift), which support the interpretation of Phase 1 alerts in relation to the error trend.

**Fig. 6:**
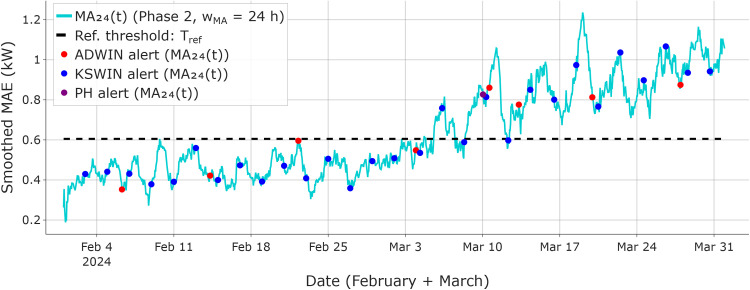
Phase 2 confirmation under incremental logarithmic drift (maximum 50 %): ADWIN, KSWIN, and PH monitor the 24-h moving-average MAE signal, with alerts overlaid on the curve. The dashed line denotes the reference threshold, computed from the drift-free reference window and used as the Phase 2 persistence boundary during the consensus interval.

Phase 2 is detailed in Fig. 6, where ADWIN, KSWIN, and PH are applied to the 24-h moving-average MAE signal $MA_{24}\left(t\right)$.

Phase 2 alerts cluster within March 6–10, completing the three-detector consensus. Over the consensus interval $\left[t_{i}^{*},\;t_{l}^{*}\right]$, $MA_{24}\left(t\right)$ remains above the reference threshold $T_{\text{ref}}$​. At the detector alert timestamps, the monitored signal is ≈0.76kW for KSWIN (March 6), rises to a local maximum of ≈0.82kW shortly thereafter, and reaches ≈0.83kW (PH, March 10) and ≈0.86kW (ADWIN, March 10). After the final alert that completes consensus (ADWIN on March 10), $MA_{24}\left(t\right)$ continues to increase and exceeds 1.0 kW later on March 10.

To preserve the original one-month training design, the retraining dataset is defined by a fixed-length window anchored at the consensus-completion time $t_{l}^{*}$​. Specifically, the retraining window is [$\;t_{l}^{*}-22$ days, $t_{l}^{*}+7$ days], which for $t_{l}^{*}=\text{March}\;10$ corresponds to February 17–March 17.

### Accumulated drift under linear drift levels

**Fig. 7:**
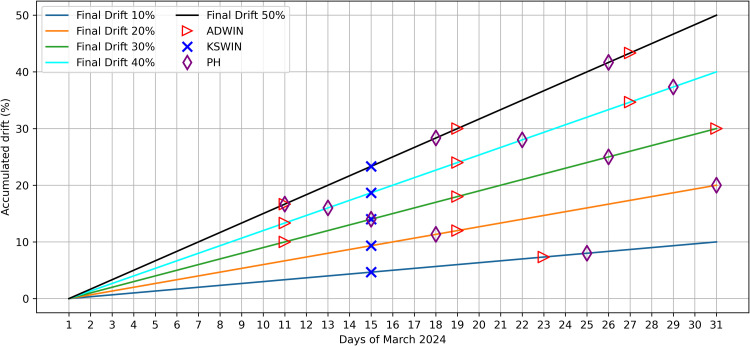
Theoretical accumulated linear-drift curves (10 %–50 %) with **Phase 1 detection-day markers** indicating the day-of-month in March when ADWIN, KSWIN, and PH triggered on the raw series.

Fig. 7 summarizes the theoretical accumulated linear-drift profiles (final levels of 10 %–50 %) and overlays the corresponding Phase 1 (raw-series) detection-day markers in March produced by ADWIN, KSWIN, and PH.

Using the fixed configuration in Table 2, the overlaid markers indicate the day-of-month positions where each detector triggered during March for each linear drift magnitude. KSWIN triggers at a fixed mid-March date (March 15) across all drift levels. In contrast, ADWIN and PH show later first alerts under lower drift (e.g., ADWIN: March 23 in the 10 % case, corresponding to ≈7.5% accumulated drift on that curve; PH: March 25 in the 10 % case) and earlier first alerts as the injected distortion increases. This monotonic advance in first-alert timing is explicit for PH (10 %: March 25; 20 %: March 18; 30 %: March 15; 40 %: March 13; 50 %: March 11) and for ADWIN (10 %: March 23; 20 %: March 19; 30 %–50 %: March 11). Additional triggers are also observed for higher drift levels (e.g., ADWIN second alerts at March 19 in the 30 %–50 % cases), and full alert sequences (including triggers beyond the first alert) for each drift level are reported in the Supplementary Material.

Overall, Fig. 7 provides a compact validation readout that, under controlled incremental linear drift, first-alert timing shifts earlier as distortion magnitude increases for ADWIN and PH, while KSWIN remains fixed under the selected configuration [21].

### Accumulated drift under logarithmic drift levels

**Fig. 8:**
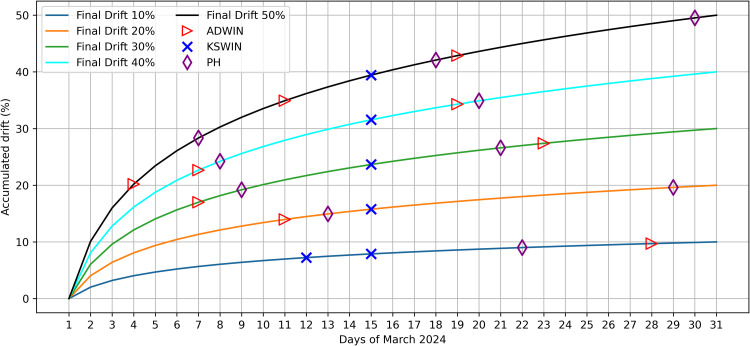
Theoretical accumulated logarithmic-drift curves (10 %–50 %) with Phase 1 detection-day markers indicating the day-of-month in March when ADWIN, KSWIN, and PH triggered on the raw series.

Fig. 8 summarizes the theoretical accumulated logarithmic-drift profiles (final levels of 10 %–50 %) and overlays the corresponding Phase 1 (raw-series) detection-day markers in March produced by ADWIN, KSWIN, and PH.

Using the fixed configuration in Table 2, the overlaid markers indicate the day-of-month positions where each detector triggered during March for each drift magnitude under logarithmic accumulation. KSWIN triggers around mid-March for most curves (≈March15), with the 10 % case triggering on March 12. ADWIN exhibits a drift-level-dependent pattern, with later detection at 10 % (March 28), earlier detection at 20 % (March 11), and higher alert density for ≥30% (30 %: March 7 and 23; 40 %: March 7 and 19; 50 %: March 4, 11, and 19).

PH produces fewer and more spaced triggers across drift levels (10 %: March 22; 20 %: March 13 and 29; 30 %: March 9 and 21; 40 %: March 8 and 20; 50 %: March 7, 18, and 30). Full alert sequences (including additional triggers beyond the first alert) for each drift level are reported in the Supplementary Material.

Overall, Fig. 8 provides a compact validation view that, under controlled logarithmic drift, first-alert timing and alert counts vary with drift magnitude and accumulation rate, which is consistent with the expected sensitivity differences among change-detection tests [21]. Phase 1 is used as a screening step on the raw stream, while Phase 2 applies the same detectors to the MAE-based monitoring signal and triggers retraining only when the persistence criterion is satisfied.

### Real-data validation

TPDM-AR was further validated on the UCI Individual Household Electric Power Consumption dataset to assess applicability on real measurements [25]. The same multi-step forecasting configuration (48-h input window; 24-h forecasting horizon) was retained, and detector hyperparameters (ADWIN, KSWIN, and PH) were kept identical to those used in the synthetic validation. After standard preprocessing (timestamp parsing, handling of missing values, and hourly resampling of active power), the MLP model was trained on July 2007 data and deployed for monitoring in August 2007.

For Phase 2, the reference threshold was calibrated from the post-deployment reference week (Aug 1–7) as ${\text{MAE}}_{\text{max}}^{\text{ref}}=\text{ma}x_{t\;\in \;\text{ref}}\;\text{MA}E_{24}\left(t\right)$, yielding $T_{\text{ref}}=0.696\;\mathrm{kW}$ (dashed line in Supplementary Fig. S4). This short calibration window is adopted because drift-free stationarity cannot be assumed over extended real-world periods; restricting calibration to the first week reduces the risk of threshold contamination by potential regime changes later in the month. During the subsequent monitoring period (Aug 8–31), Phase 2 alerts were observed on the smoothed monitoring signal $MA_{24}\left(t\right)$. In Supplementary Fig. S4, $MA_{24}\left(t\right)$ exceeded $T_{\text{ref}}$ only in two short intervals (Aug 19–20 and Aug 24–25); only one Phase 2 alert occurred while $MA_{24}\left(t\right)$ was above $T_{\text{ref}}$ (KSWIN on Aug 20), and no PH alerts were recorded during the monitoring period. However, retraining was not triggered because the Phase 2 confirmation rule was not satisfied: three-detector consensus within the consensus horizon S and sustained exceedance of $T_{\text{ref}}$ over the resulting interval $\left[t_{i}^{*},\;t_{l}^{*}\right]$​ were not jointly met.

This outcome is consistent with TPDM-AR’s conservative retraining logic: transient error excursions may raise Phase 2 alerts, but retraining is executed only when the joint consensus-and-persistence confirmation is satisfied, reducing unnecessary retraining. Therefore, no Phase 2 consensus-and-persistence confirmation was achieved in August 2007, and no retraining action was scheduled for this monitoring cycle. For completeness, Phase 1 monitoring outputs for the same period are shown in Supplementary Figs. S3 and S5; in the raw active-power stream, all three detectors emitted alerts during August monitoring (KSWIN: 10 alerts, ADWIN: 2, PH: 1), but these raw-stream shifts were not jointly confirmed as sustained predictive degradation in Phase 2.

A compact overview combining hourly active power, the daily mean $\text{MA}E_{24}\left(t\right)$ trend, Phase 1 triggers, and the reference threshold is shown in Supplementary Fig. S6. The consolidated real-data outputs (Phase 1 and Phase 2 summaries, trigger timestamps, and exported metrics) are reported in Supplementary Table S2 (multi-sheet workbook).

## Limitations

The present study is intended as a proof-of-concept validation of the TPDM-AR workflow under controlled conditions. Accordingly, it does not benchmark the proposed framework against alternative drift-handling strategies, such as single-detector baselines, weighted detector ensembles, or adaptive threshold schemes. This is consistent with the paper's focus on establishing the viability of the proposed workflow rather than demonstrating superiority over competing approaches. A systematic comparison across these alternatives, including an evaluation of detection delay, false-positive rate, and retraining frequency, is identified as a direct next step and constitutes a natural extension of this methodology paper.

The synthetic evaluation uses linear and logarithmic incremental drift profiles as controlled distortions. Although real-world drift can be more complex, involving abrupt changes, gradual transitions with recovery, or recurrent concept re-emergence, the controlled profiles used here were deliberately chosen to provide a reproducible testbed for transparent evaluation of detector sensitivity and detection delay under precisely known conditions. This approach is consistent with standard practice in the drift detection literature [3,14] and allows the proposed two-phase decision logic to be assessed before deployment on real measurements. The simplicity of the drift profiles is therefore a methodological choice rather than a claim of full operational realism. Evaluation under more complex drift types is identified as a priority for future work.

Only three detectors, namely ADWIN, KSWIN, and PH, were evaluated, as they are suitable for regression and continuous data streams. Other detectors, such as DDM or EDDM, were excluded because they are more directly associated with classification-error settings and class-label-driven monitoring. The effectiveness of TPDM-AR also depends on domain knowledge to define operationally meaningful reference behavior and acceptable degradation levels.

This study evaluated TPDM-AR using a single forecasting model (MLPRegressor) as a canonical regression baseline. The TPDM-AR architecture is model-agnostic, since the two-phase drift monitoring and retraining logic operates independently of the forecasting model, and any scikit-learn-compatible regressor can replace the MLP without modifying the monitoring pipeline. The use of a single model is therefore a deliberate simplification intended to isolate the behavior of the drift detection workflow. It does not imply that the framework is specific to MLP-based regressors. Evaluation with additional model classes, including gradient-boosted trees, support vector regression, and LSTM-based architectures, as well as additional datasets, is planned as future work.

In real-data operation, Phase 2 confirmation is inherently delayed by the need to observe the full 24-step-ahead error horizon required to compute the horizon-aggregated monitoring signal $\text{MA}E_{24}\left(t\right)$.

Several directions are identified for future work. First, the evaluation of TPDM-AR under more complex drift profiles, including abrupt, gradual, and recurrent drift, would broaden the empirical evidence beyond the incremental scenarios studied here. Second, assessing the framework with alternative regression architectures, such as gradient-boosted trees, support vector regression, and LSTM-based models, would allow investigation of its generalisability across model classes. Third, a systematic sensitivity analysis of the key hyperparameters, namely ADWIN delta, KSWIN window size and significance level, Page–Hinkley threshold and alpha, and the consensus horizon S, is planned to provide guidance on domain-specific calibration. Finally, deployment of TPDM-AR in a live smart-campus environment, operating on real-time metered data, is the intended next step toward operational validation.

## Ethics statements

Not applicable. This study did not involve human participants or animals, and no social media data were used.

## Supplementary material *and/or* additional information

The Supplementary Material accompanying this manuscript contains the code and finalized supplementary files (edited figures, consolidated tables, the Supplementary Synthetic Dataset, interactive HTML exports, and environment files) used to reproduce and document the reported analyses and outputs. The materials are organized as follows (a file-by-file description with a descriptive caption per item is provided in the Supplementary Material README.md):

• Code & environment: Code and environment files are provided in the Supplementary Material, including the main notebook/script (00_main_pipeline.ipynb), requirements.txt, requirements-lock.txt, and README.md. The 00_main_pipeline.ipynb notebook reproduces the complete workflow and the reported outputs.
• Supplementary Synthetic Dataset (CSV, baseline Jan–Mar 2024): synthetic_baseline_jan_feb_mar_2024.csv.
• Interactive visualizations (HTML, real-world validation; Plotly exports): phase1_raw.html, phase2_mae.html, phase1_power_vs_pred.html, and phase1_power_vs_daily_mae.html.
• Supplementary Figure S1 (synthetic, linear drift 50 %): ADWIN, KSWIN, and Page–Hinkley (PH) alerts on actual vs. predicted hourly average active power. File: Supplementary_Figure_S1.png.
• Supplementary Figure S2 (synthetic, logarithmic drift 50 %): ADWIN, KSWIN, and Page–Hinkley (PH) alerts on actual vs. predicted hourly average active power. File: Supplementary_Figure_S2.png.
• Supplementary Figure S3 (real-world, Phase 1, Aug 2007): drift detection on raw hourly average active power with detector alerts. File: Supplementary_Figure_S3.png.
• Supplementary Figure S4 (real-world, Phase 2, Aug 2007): MAE-based monitoring using 24-step forecasts (48-h input, 24-h horizon), showing $MA_{24}\left(t\right)$ and $\text{MA}E_{24}\left(t\right)$ with detector alerts and the reference threshold $T_{\text{ref}}$. File: Supplementary_Figure_S4.png.
• Supplementary Figure S5 (real-world, Phase 1, Aug 2007): hourly average active power vs. MLP forecast (t+1 extracted from the 24-step forecast), with detector alerts. File: Supplementary_Figure_S5.png.
• Supplementary Figure S6 (real-world, Phase 1 + Phase 2 context, Aug 2007): hourly average active power vs. daily mean $\text{MA}E_{24}\left(t\right)$ with Phase 1 drift triggers and the reference threshold ${\text{MAE}}_{\text{max}}^{\text{ref}}$ (48-h input, 24-h horizon). File: Supplementary_Figure_S6.png.
• Supplementary Tables S1–S2 (consolidated multi-sheet Excel workbooks):
• S1 (synthetic experiments): Table_S1_Synthetic.xlsx. Sheets Lin10–Lin50 and Log10–Log50 report daily mean MAE/MSE/RMSE for February (no drift) vs. March (drift), together with the corresponding Daily Drift ( %), plus a detection-day summary, accumulated drift curves, and a Config sheet with experiment settings.
• S2 (real-world experiments, UCI Aug 2007): Table_S2_UCI.xlsx. The workbook includes daily mean and hourly $\text{MA}E_{24}\left(t\right)$, $\text{MS}E_{24}\left(t\right)$, and $\text{RMS}E_{24}\left(t\right)$; exact plotted trigger points for Phase 1 and Phase 2; and a consensus sheet with $\left[t_{i}^{*},\;t_{l}^{*}\right]$ and the reference threshold ${\text{MAE}}_{\text{max}}^{\text{ref}}$.
• Real-world dataset (UCI, ID 235): not included. On first run, 00_main_pipeline.ipynb downloads/extracts the raw file and regenerates the hourly dataset cache as data/uci_household_power_JulAug2007_hourly_interp.csv; see README.md for the offline/local-file option.

## Declaration of generative AI and AI-assisted technologies in the manuscript preparation process

During the preparation of this work, the authors used ChatGPT (OpenAI) for translation from Portuguese to English, language polishing, and review of analysis scripts. No AI tools were used to generate the scientific results, data analyses, figures, tables, or conclusions. All technical content was verified by the authors, who take full responsibility for the manuscript.

## Related research article

None.

## CRediT authorship contribution statement

**Michel B. Oliveira:** Methodology, Software, Validation, Formal analysis, Investigation, Data curation, Writing – original draft, Writing – review & editing, Visualization. **Leandro A. Silva:** Conceptualization, Validation, Resources, Writing – review & editing, Supervision, Project administration, Funding acquisition.

## Declaration of competing interest

The authors declare that they have no known competing financial interests or personal relationships that could have appeared to influence the work reported in this paper.

## Data Availability

Code, synthetic baseline data, and supporting files are provided as Supplementary Material. UCI Household Power Consumption data (ID 235) is public and not redistributed (see README).

## Appendix Supplementary materials

Image, application 1

Image, application 2

Image, application 3

Image, application 4

Image, application 5

Image, application 6

Image, application 7

Image, application 8

Image, application 9

Image, application 10

Image, application 11

Image, application 12

Image, application 13

Image, application 14

Image, application 15

Image, application 16

Image, application 17
