# Supplementary figures and images for: Two-phase drift monitoring and automatic retraining (TPDM-AR) for electric load forecasting
Source: MethodsX. 2026 May 20;16:103966. doi: 10.1016/j.mex.2026.103966 (PMC13226265; doi:10.1016/j.mex.2026.103966)

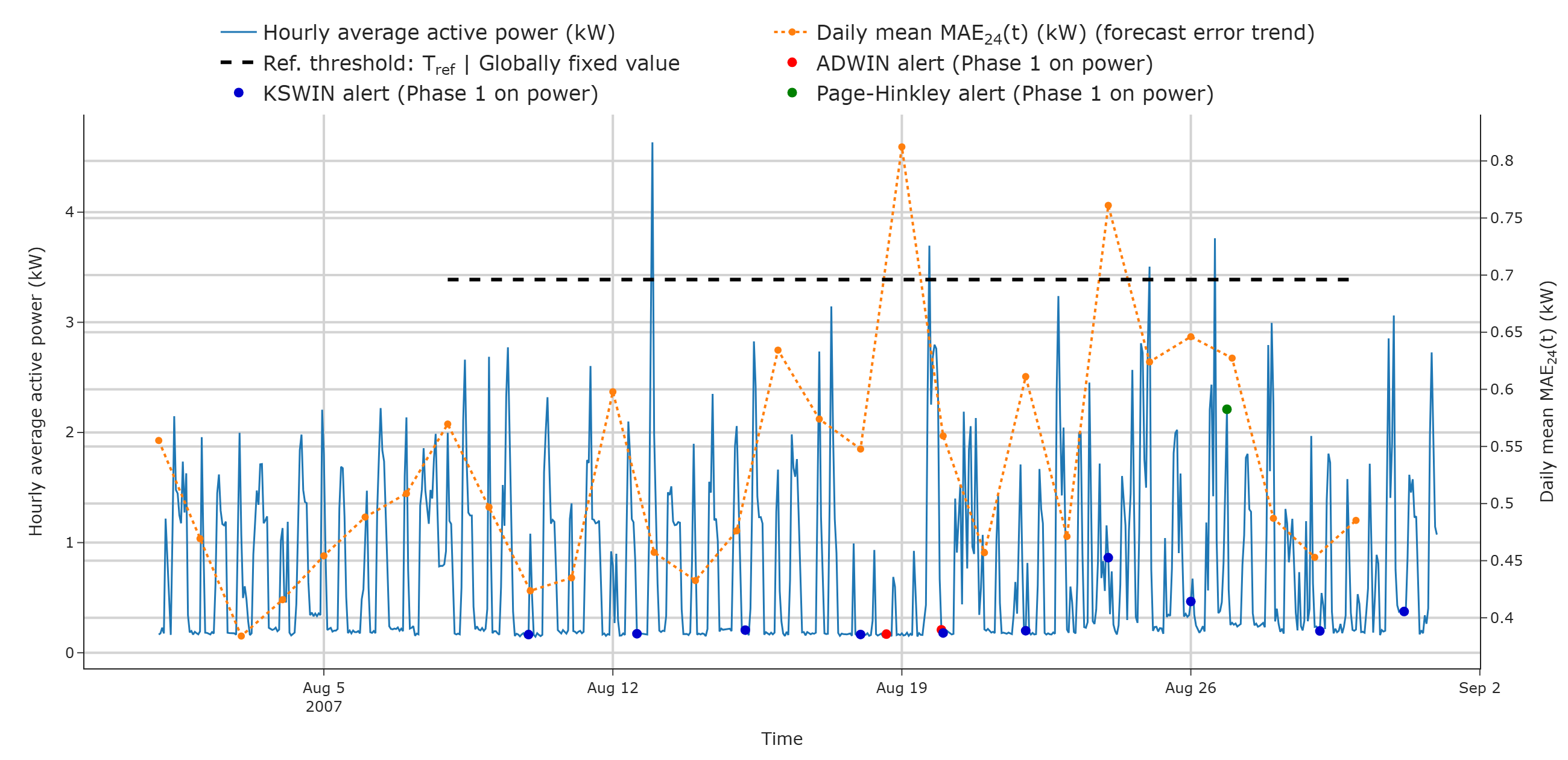

Supplement: Supplementary file 3 [file mmc3.zip › mmc3.png]

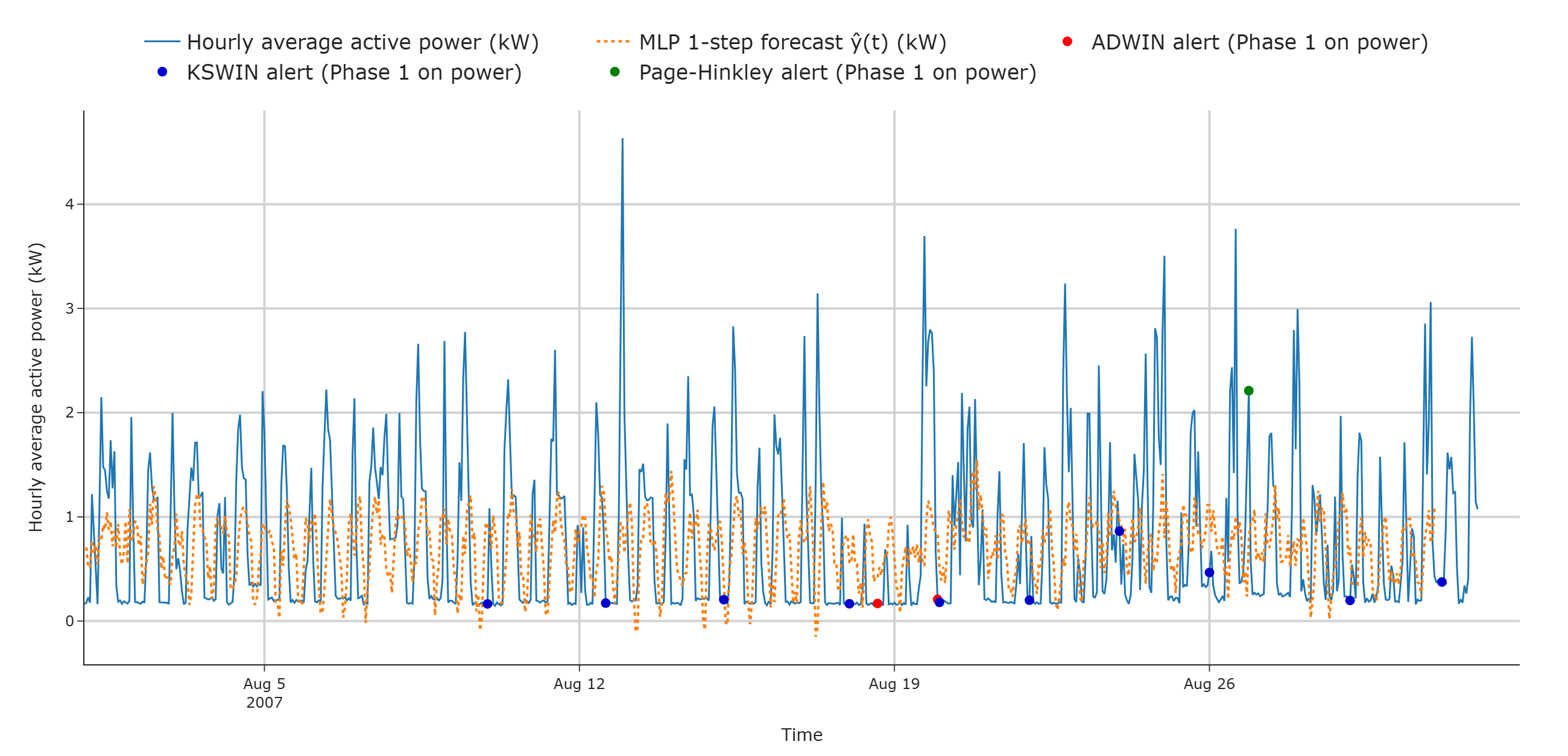

Supplement: Supplementary file 4 [file mmc4.zip › mmc4.png]

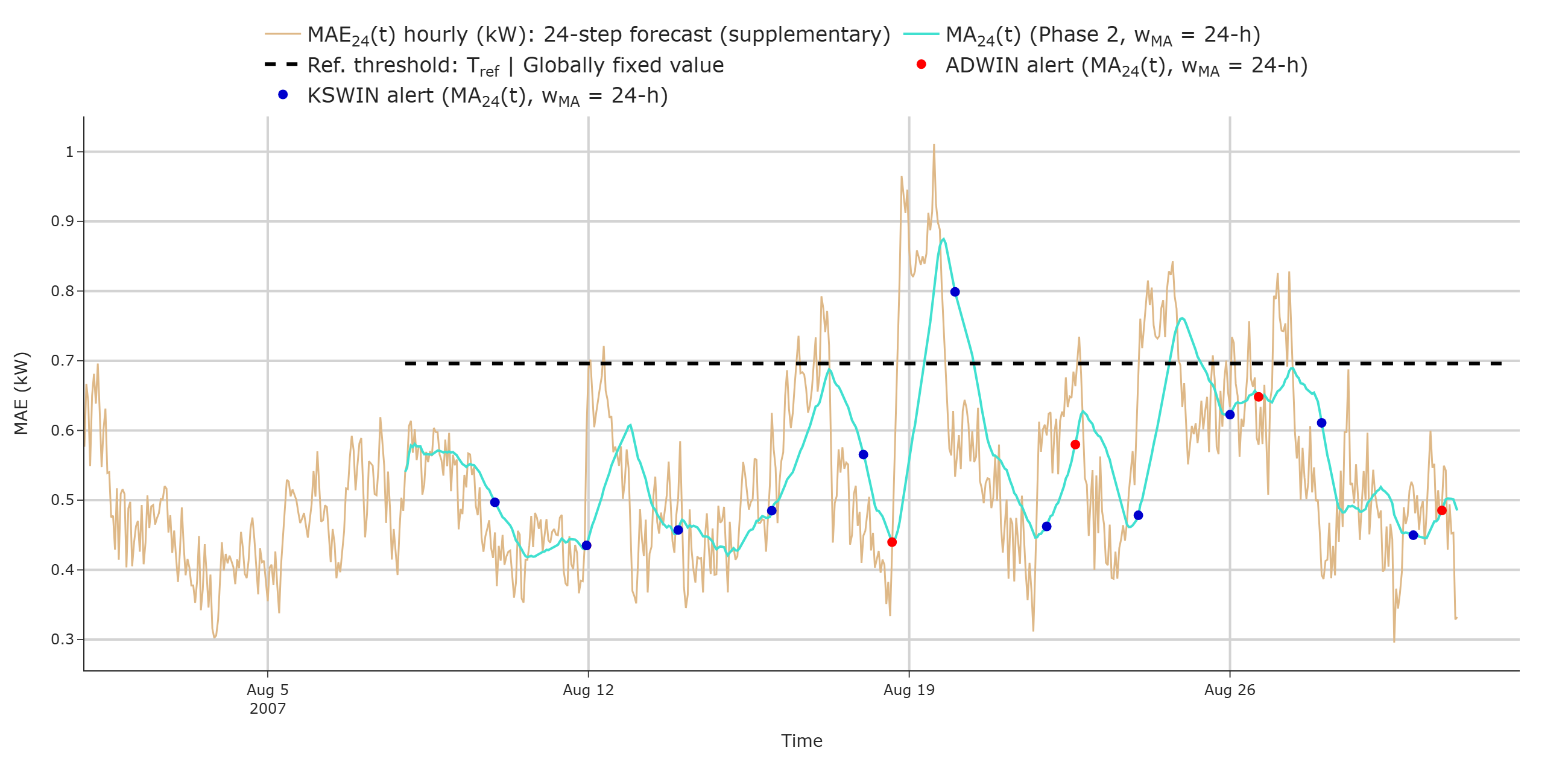

Supplement: Supplementary file 5 [file mmc5.zip › mmc5.png]

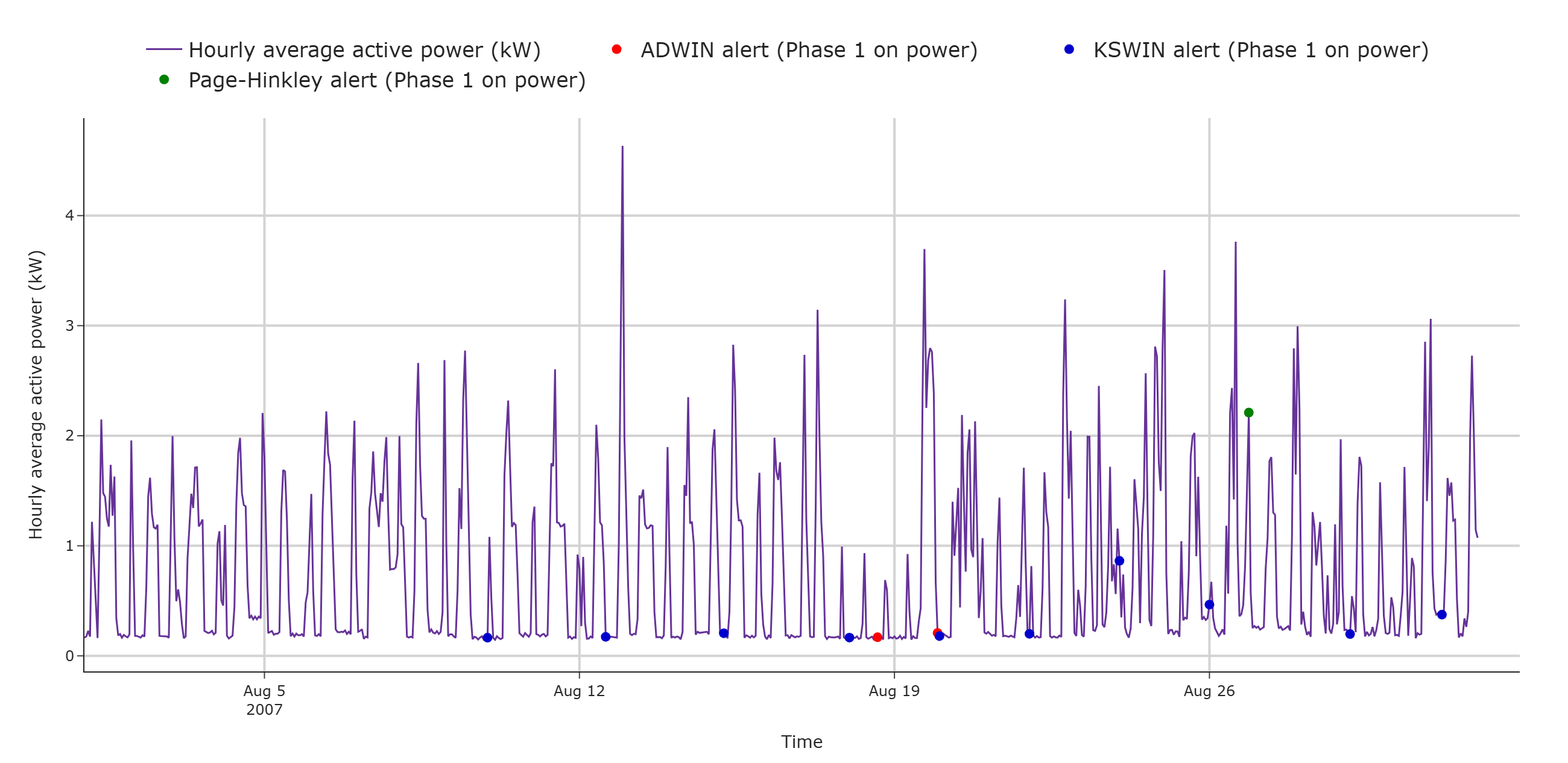

Supplement: Supplementary file 6 [file mmc6.zip › mmc6.png]

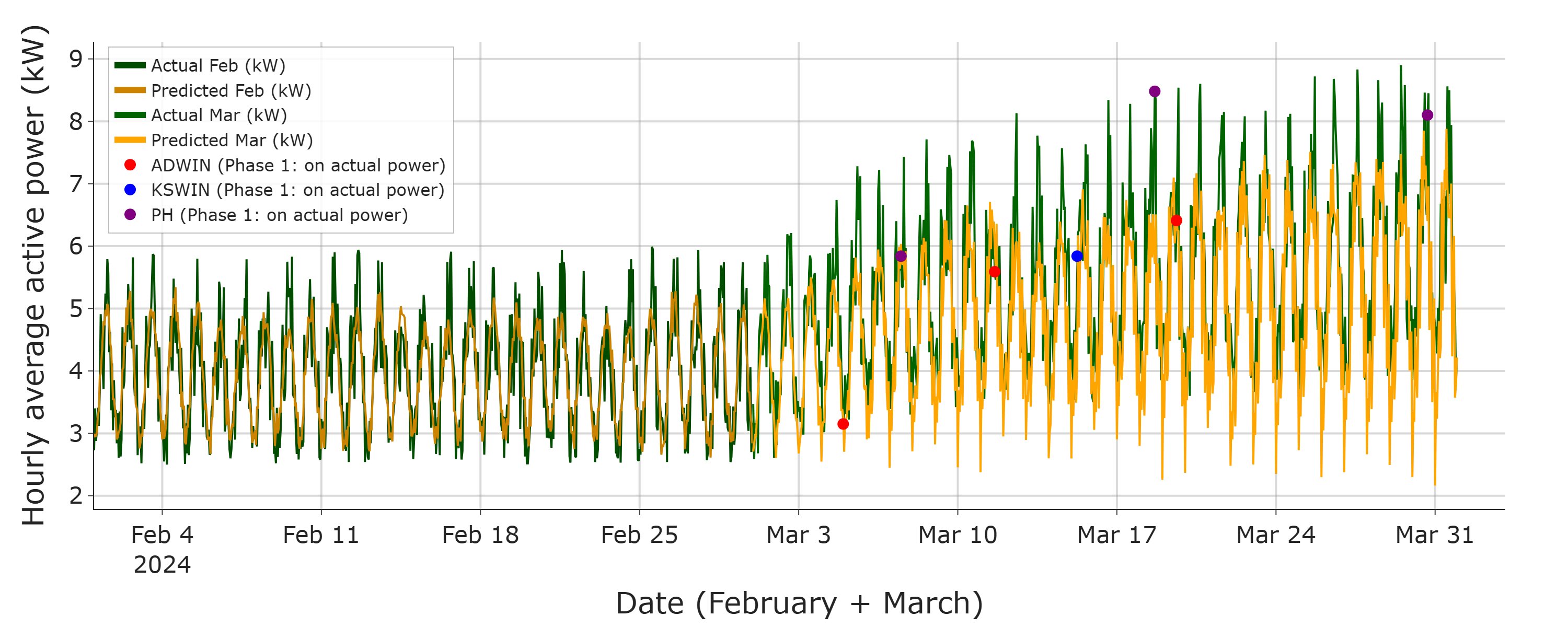

Supplement: Supplementary file 7 [file mmc7.zip › mmc7.png]

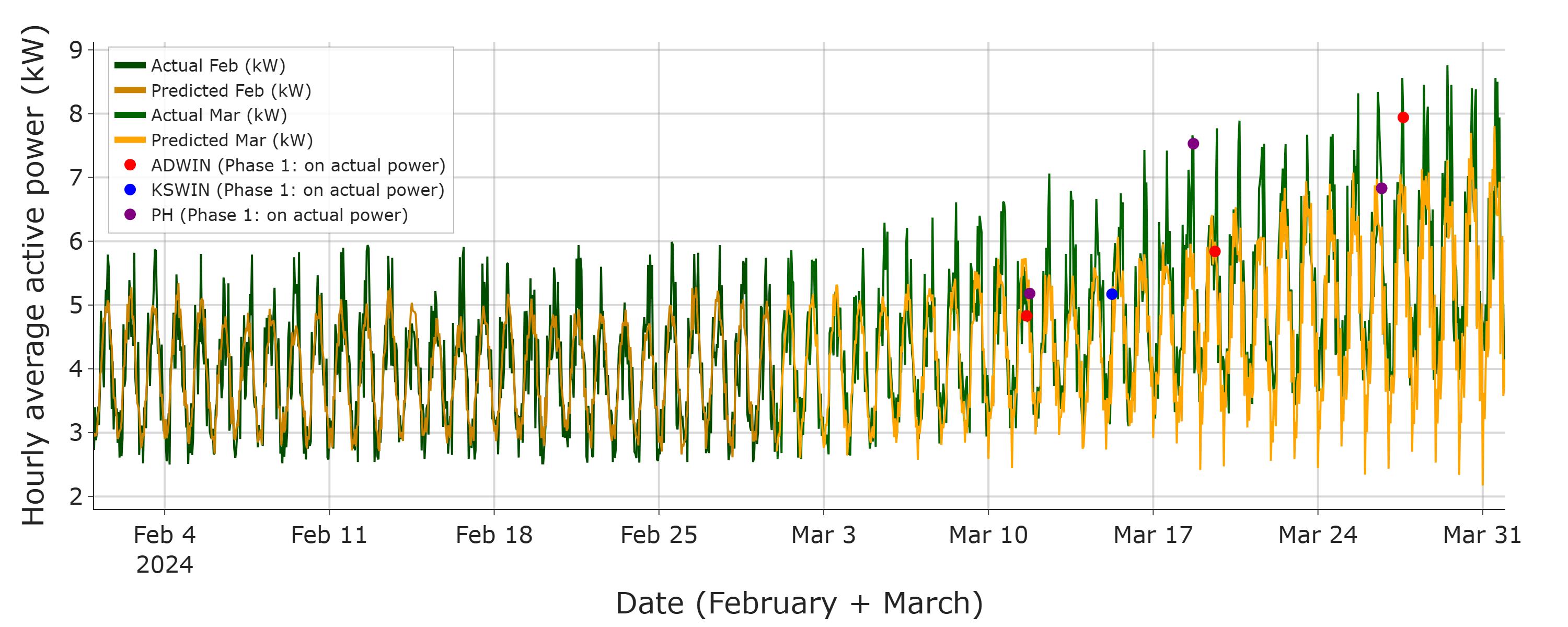

Supplement: Supplementary file 8 [file mmc8.zip › mmc8.png]
